# IFT88 influences chondrocyte actin organization and biomechanics

**DOI:** 10.1016/j.joca.2015.10.003

**Published:** 2016-03

**Authors:** Z. Wang, A.K.T. Wann, C.L. Thompson, A. Hassen, W. Wang, M.M. Knight

**Affiliations:** † Institute of Bioengineering and School of Engineering and Materials Science, Queen Mary University of London, London, United Kingdom; ‡ Kennedy Institute of Rheumatology, University of Oxford, Oxford, United Kingdom

**Keywords:** ITF88, F-actin, Myosin IIB, Cell mechanics, Micropipette aspiration

## Abstract

**Objectives:**

Primary cilia are microtubule based organelles which control a variety of signalling pathways important in cartilage development, health and disease. This study examines the role of the intraflagellar transport (IFT) protein, IFT88, in regulating fundamental actin organisation and mechanics in articular chondrocytes.

**Methods:**

The study used an established chondrocyte cell line with and without hypomorphic mutation of IFT88 (IFT88^orpk^). Confocal microscopy was used to quantify F-actin and myosin IIB organisation. Viscoelastic cell and actin cortex mechanics were determined using micropipette aspiration with actin dynamics visualised in live cells transfected with LifeACT-GFP.

**Results:**

IFT88^orpk^ cells exhibited a significant increase in acto-myosin stress fibre organisation relative to wild-type (WT) cells in monolayer and an altered response to cytochalasin D. Rounded IFT88^orpk^ cells cultured in suspension exhibited reduced cortical actin expression with reduced cellular equilibrium modulus. Micropipette aspiration resulted in reduced membrane bleb formation in IFT88^orpk^ cells. Following membrane blebbing, IFT88^orpk^ cells exhibited slower reformation of the actin cortex. IFT88^orpk^ cells showed increased actin deformability and reduced cortical tension confirming that IFT regulates actin cortex mechanics. The reduced cortical tension is also consistent with the reduced bleb formation.

**Conclusions:**

This study demonstrates for the first time that the ciliary protein IFT88 regulates fundamental actin organisation and the stiffness of the actin cortex leading to alterations in cell deformation, mechanical properties and blebbing in an IFT88 chondrocyte cell line. This adds to the growing understanding of the role of primary cilia and IFT in regulating cartilage biology.

## Introduction

The primary cilium is a single hair-like structure expressed by the majority of mammalian cell types. The cilium consists of a basal body which originates from the mother centriole and extends the ciliary axoneme which is formed by an array of nine acetylated microtubule doublets surrounded by a specialised membrane. Primary cilia are involved in regulating various fundamental cell signalling pathways including Wnt, hedgehog, PCP, growth factor signalling and mechanotransduction (for review see^1^). These pathways are highly influential in controlling cellular patterning and matrix organisation during the development of limbs and joints (for review see^2^, ^3^). Cilia signalling pathways rely on the trafficking of proteins into and out of the cilium by a collection of specialised intraflagellar transport (IFT) proteins and molecular motors. Disruption of IFT proteins therefore interferes with signalling and the maintenance of cilia structure, frequently resulting in stunted or absent primary cilia (for review see^4^).

In various cell types, the mutation of primary cilia proteins has been reported to affect fundamental actin organisation^5^, ^6^. Previous studies have also shown that intracellular tension and the organisation of the actin cytoskeleton can regulate the expression of primary cilia through the control of cilia trafficking^7^, ^8^. This indicates the existence of a complex, reciprocal relationship between IFT and cytoskeletal structures which may influence fundamental cell behaviour, although the details remain poorly understood.

In the developing cartilage, McGlashan *et al.* observed altered actin organisation in chondrocytes *in situ* following loss of function of the ciliary protein IFT88, known as the Oak Ridge Polycystic Kidney disease (ORPK) model^9^. This was supported by similar observations in KIF3A null mice^10^. The current study therefore focusses on articular chondrocytes which express primary cilia both in the developing growth plate^9^, ^10^, ^11^, ^12^ and in adult articular cartilage^13^, ^14^, ^15^, ^16^ where they regulate cartilage development, health and disease^9^, ^10^, ^12^, ^17^, ^18^, ^19^, ^20^, ^21^, ^22^, ^23^, ^24^, ^25^, ^26^. In particular, previous studies have shown that hypomorphic mutation of IFT88 (IFT88^orpk^) prevents chondrocyte mechanotransduction^27^ and disrupts the development of articular cartilage leading to osteoarthritic-like tissue^28^ with reduced mechanical integrity^29^. Furthermore, several of these cilia signalling pathways have been implicated in the pathogenesis of osteoarthritis^24^, ^25^, ^26^.

The present study therefore tests the hypothesis that IFT88 directly regulates chondrocyte actin cytoskeletal organisation and biomechanics. The results demonstrate for the first time, that IFT88 directly regulates F-actin/myosin organisation and dynamics and the associated mechanical properties of the actin cortex in articular chondrocytes. This leads to alterations in cellular deformability, equilibrium modulus and bleb formation. The study adds to a growing understanding of the role of primary cilia and associated proteins, known as the ciliome, in regulating fundamental aspects of cell structure and function important in health and disease.

## Methods

### Cell culture

Conditionally immortalized wild-type (WT) and mutant IFT88^orpk^ mouse chondrocyte cell lines were generated as described previously^27^. The IFT88^orpk^ chondrocytes harbour an insertional hypomorphic mutation to the gene tg737, encoding for the anterograde intraflagellar trafficking protein IFT88, resulting in strong inhibition of primary cilia assembly. Chondrocytes were maintained in DMEM supplemented with 10% FCS, 88 U/ml penicillin, 90 μg/ml streptomycin, 10 ng/ml INF-γ and 2.5 mM L-glutamine (Sigma–Aldrich, Poole, UK). Immortalized cells were maintained under permissive conditions at 33°C, 5% CO_2_ in the presence of 10 nM IFN-γ. For experiments, cells were cultured in non-permissive conditions at 37°C (without IFN-γ) for 3 d, to switch off SV40 control and restore the primary chondrocyte phenotype, then seeded in 24 wells plate for another 24 h. Cell viability was assessed with trypan blue.

### Quantification of actin-myosin organisation in monolayer and the effect of cytochalasin D

WT and IFT88^orpk^ cells were cultured as a monolayer on glass coverslips for 24 h and then fixed with 4% paraformaldehyde (PFA) for 10 min. Cells were incubated with primary antibodies at room temperature for 4 h. Mouse monoclonal anti-acetylated tubulin, clone 611B-1 (Sigma Aldrich) was used at 1:2000 for the detection of the ciliary axoneme. Rabbit monoclonal anti-non-muscle myosin IIA, clone EPR8965 and rabbit monoclonal anti-non-muscle myosin IIB, clone 3H2 (both Abcam, Cambridge, UK) were used at 1:250. Following repeated washing in phosphate buffered saline (PBS), cells were incubated with appropriate Alexa 488 and Alexa 594 conjugated secondary antibodies (Molecular Probes, Paisley, UK) for 1 h at room temperature. F-actin was stained with 25 μl/ml Alexa Fluor 555-phalloidin (Molecular Probes) for 30 min at room temperature. Nuclei were labelled with 1 μg/ml 4′, 6-diamidino-2-phenylinodole (DAPI; Molecular Probes). Samples were imaged using a Leica TCS SP2 confocal microscope with a 63×, 1.3-NA lens (Leica Microsystems, Wetzlar, Germany). Confocal Z-stacks were obtained throughout the entire cellular profile with a Z-step size of 0.25 μm and an image format of 512 × 512 pixels. This produced an *xy* pixel size of 0.465 μm × 0.465 μm. Z-stacks were reconstructed and *xy* maximum intensity projections generated. Mean fluorescence intensity for F-actin and myosin was measured for individual cells using Image J software.

For cytochalasin D experiments, cells were treated with 6 μM cytochalasin D (Santa Cruz Biotechnology, Santa Cruz, CA) for 10 min, washed with fresh medium and allowed to recover for 45 min. Cells were fixed with 4% PFA followed by immunofluorescence labelling with anti-non-muscle myosin IIB and Alexa Phalloidin 555 as described above. Samples were imaged using a Leica DMI400B Epi-fluorescence Microscope with a 63×, 1.25-NA lens (Leica Microsystems) producing an *xy* pixel size of 0.146 μm × 0.146 μm. The same imaging setting were used throughout and none of the images reached intensity saturation. Images were analysed for mean cellular fluorescence F-actin intensity using Image J software.

### Measurement of RhoA activity

RhoA activity was determined for cells in monolayer using the G-LISA RhoA Activation Assay Biochemical Kit (BK124-S, Cytoskeleton Inc., Denver, CO) according to the manufacturer's protocol. In brief, this assay kit contains a Rho-GTP-binding protein which is bound to the wells of a 96 well plate. Cell lysate is applied to these wells and active, GTP-bound RhoA becomes bound and can be quantified while inactive GDP-bound RhoA is removed by washing. Cells were lysed using the manufacturer's cell lysis and protein concentration was quantified using the precision red protein assay reagent by measuring the absorbance at 600 nm. Lysates were then diluted to 0.5 mg/ml using lysis buffer and loaded on to G-LISA plate for protein analysis. Absorbance was read at 490 nm.

### Quantification of cortical F-actin organisation in rounded cells

Trypsinized cells were kept in suspension for 45 min and then fixed with 3% glutaraldehyde (Agar Scientific, Stansted, UK) for 30 min at room temperature. Cells were stained with Alexa Fluor 555-phalloidin for 30 min at 37°C. Nuclei were labelled with DAPI. Cells were imaged using the confocal microscope with a 63×, 1.3-NA lens to produce an *xy* pixel size of 0.09 μm × 0.09 μm. Mean fluorescence intensity of individual cells were measured using Image J software. F-actin spatial distribution was quantified using a method similar to previous studies^30^. An individual linear intensity profile was drawn across each cell avoiding the nucleus. The intensity of cortical F-actin (I_cortical_) was considered to be the average of the highest two intensities at the edge of the cell. The intensity of cytoplasmic F-actin (I_cytoplasmic_) was the average intensity of a 5 μm long section between these two points. Thus cortical F-actin ratio was calculated as (I_cortical_ − I_cytoplasmic_)/(I_cortical_ + I_cytoplasmic_).

### Micropipette aspiration

Micropipette aspiration was used to determine the viscoelastic properties (instantaneous and equilibrium modulus) and cortical tension of individual cells. The micropipette aspiration system is similar to that previously described^31^. The ratio of cell diameter to micropipette diameter was maintained between 2.5 and 3.3 as required by the analytical model used to calculate cell moduli^32^. Approximately 0.5 ml of cell suspension was placed in a custom built chamber on the inverted stage of a confocal microscope (Perkin Elmer, London, UK) with a ×60, 1.4-NA lens.

For measurement of viscoelastic properties, a step pressure of 7 cm of water (0.689 kPa) was applied in 1.3 s using a PC-controlled pump and LabView control system. Following the step aspiration pressure, sequential bright-field images of the cell were recorded at 1 frame every 2.15 s for 180 s. This produced an *xy* pixel size of 0.11 μm × 0.11 μm. All imaging was conducted at room temperature.

The cell volume during micropipette aspiration was calculated based on the following equation where L is the aspiration length into the micropipette.

(1) $$\text{V}=\frac{1}{6}\pi D_{h}^{2}D_{v}+\frac{1}{4}\pi D_{p}^{2}\left(L-\frac{D_{p}}{2}\right)+\frac{2}{3}\pi {\left(\frac{D_{p}}{2}\right)}^{3}$$

D_h_ and D_v_ represent the horizontal and vertical diameters of the portion of the cell outside of the micropipette which is assumed to be an oblate ellipsoid. D_p_ is the internal diameter of the micropipette and L is the aspiration length. The leading edge of the cell within the micropipette was assumed to be hemispherical with a diameter equal to that of the micropipette.

The viscoelastic parameters were calculated using the standard linear solid (SLS) model described by Sato *et al.*^33^ Three viscoelastic parameters, k_1_, k_2_ and μ were determined by fitting the following equation using nonlinear regression analysis.

(2) $$L\left(t\right)=\frac{Øa\Delta P}{\pi k1}\left[\frac{1-\left(\frac{k_{1}}{k_{1}\;+\;k_{2}}-1\right)}{e^{-t/\tau }}\right]$$

(3) $${\text{E}}_{\text{in}}=\frac{3}{2}\left(k_{1}+k_{2}\right)$$

(4) $${\text{E}}_{\text{eq}}=\frac{3}{2}k_{1}$$

(5) $$\mu =\tau \left(\frac{k_{1}k_{2}}{k_{1}\;+\;k_{2}}\right)$$

L(t) is the aspiration length of the cell at time t; E_in_ and E_eq_ represent the instantaneous moduli and equilibrium moduli respectively; μ is the apparent viscosity; and τ is the time constant. Ø is defined as the wall function with a value of 2.1 for the micropipettes used in this study^34^. Cells were rejected from the analyses if they did not aspirate, were completely aspirated or the model curve fit R^2^ value was less than 0.9 (see Table I).

For measurement of cortical tension, an experimental protocol similar to previous studies was used^35^. In brief, an individual cell was subjected to negative pressure in a series of 14 increments of 0.5 cm of water (0.049 kPa) every 15 s to a maximum pressure of 7 cm of water (0.689 kPa). As the pressure was gradually increased, a critical threshold pressure ΔP was reached, at which the aspirated portion of the cell formed a hemispherical protrusion such that the aspirated length, L, equalled the inner micropipette radius, R_p_. The cortical tension, T, was calculated using the following equation based on the liquid-drop model as previously described^36^.

(6) $$\text{T}=\frac{\Delta {\text{P}}_{c}}{2\left(\frac{1}{R_{P}}\;-\;\frac{1}{R_{C}}\right)}$$

where R_c_ is the radius of the cell outside of the micropipette.

### Visualisation of F-actin dynamics in live cells

In order to image the dynamics of cortical F-actin, cells were transduced with an adenovirus delivering a proven F-actin marker, rAV^CMV^-LifeAct-TagGFP2 (IBDI, Verona, WI). Virus particles were added to the cells at a pre-optimised multiplicity of infection (MOI) according to manufacturer's protocol. After 36 h incubation (37°C, 5% CO_2_), cells were washed with fresh medium and prepared in suspension for micropipette aspiration.

### Data analyses

All statistical analyses were conducted using the software SPSS (Ver. 13.0, SPSS Inc., Chicago, IL, USA). An experiment is defined as a single passage of the cells. For G-LISA RhoA activation assay, data was collected from three passages of cells. The rest of experiments were performed at least twice. Normality testing (Shapiro-Wilk test) was performed for all experimental data. For parametric statistics, data was presented as mean with 95% confidence interval (CI) and assessed by unpaired Student's *t* test. For non-parametric statistics, data was presented as the median and interquartile range and assessed by Mann–Whitney *U* test. Chi-square test was used to examine the differences between two proportions. For analysis of the rate of fluorescence intensity recovery, mean intensity from individual time points were plotted over time and fitted using linear regression. The slope of a fitted line represented the relative change in intensity (%) with time (min). Univariate analysis of variance was used to examine the differences between two slopes. In all cases, 2-tailed tests were employed. Differences were considered statistically significant at *P* < 0.05.

## Results

### IFT88 mutation increases F-actin and non-muscle myosin IIB (NMIIB) expression in monolayer

**Fig. S1:**
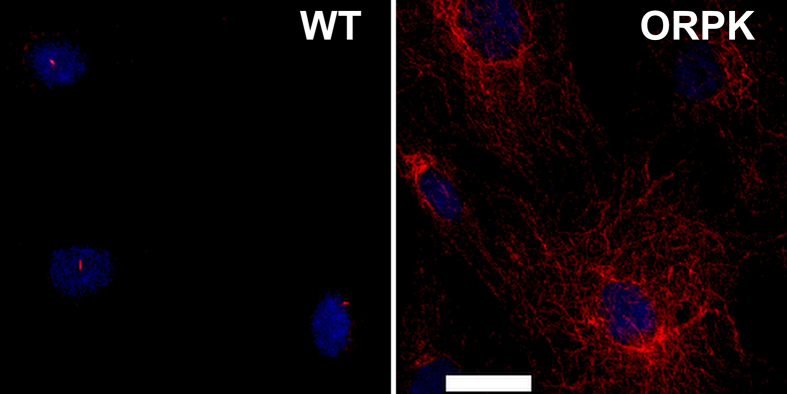
Representative confocal maximum intensity Z projections showing primary cilia immuno-fluorescently labelled in WT and IFT88^orpk^ chondrocytes in sub-confluent culture. The cilium was labelled with antibodies directed to acetylated α-tubulin (red) while nuclei were counterstained with DAPI (blue). Scale bar represents 25 μm.

**Fig. 1:**
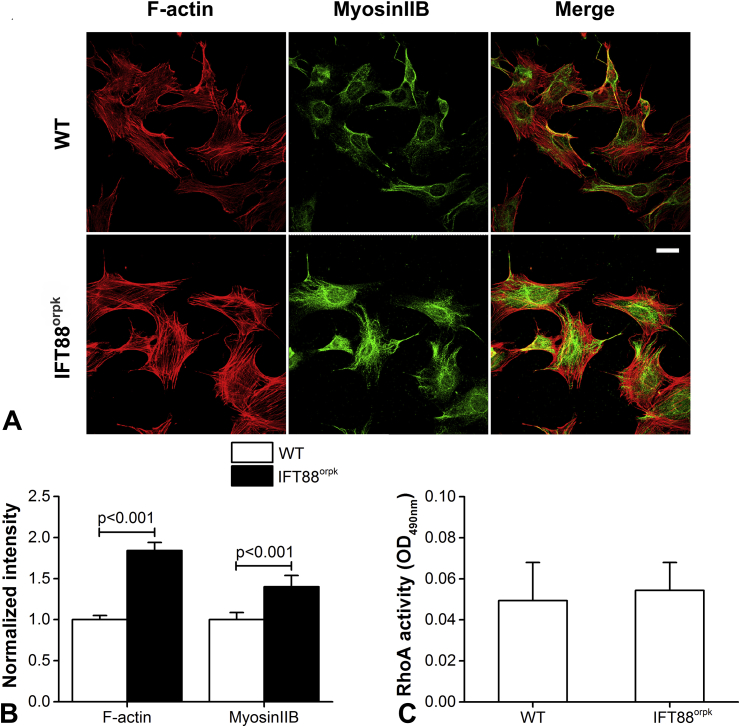
**IFT88 mutation increases F-actin and non-muscle myosin IIB expression independent of RhoA**. (A) Representative confocal maximum intensity Z projections showing F-actin and myosin IIB immuno-fluorescently labelled for WT and IFT88^orpk^ cells in sub-confluent cultures. F-actin was stained with Alexa Fluor 555-phalloidin (red) while myosin IIB was immuno-fluorescently labelled for NMIIB (green). Scale bar represents 25 μm. (B) Quantification of F-actin and myosin IIB for WT and IFT88^orpk^ cells in sub-confluent cultures. Fluorescence intensities were quantified from epifluorescence images and normalised to the mean intensity in WT cells. Data represents mean ± CI (*N* = 32 and 36). Data was analysed by unpaired Student's *t* test. (C) RhoA activity for WT and IFT88^orpk^ cells in sub-confluent cultures. RhoA activity was measured using a RhoA G-LISA assay. Data represents mean ± CI (*N* = 9 and 10). Data was analysed by unpaired Student's *t* test.

Immunofluorescence staining was used to investigate the existence of primary cilia and organisation of F-actin cytoskeleton for WT and IFT88^orpk^ chondrocytes in sub-confluent cultures. The absence of cilia and greatly increased level of cytoplasmic acetylated α-tubulin in IFT88^orpk^ chondrocytes confirmed the disruption of ciliogenesis caused by IFT88 mutation (Fig. S1). For both cell types, well aligned stress fibres composed of F-actin and NMIIB were clearly observed [Fig. 1(A)]. Interestingly, NMIIB was mainly found in a perinuclear location. Myosin IIA was not detected in either cell type (data not shown). IFT88^orpk^ cells showed significantly increased levels of F-actin and NMIIB staining compared to WT cells (*P* < 0.001 in both cases; Fig. 1(B)). We also examined RhoA activity using a G-LISA RhoA activation kit, but found no differences between the two cell types (*P* = 0.683; Fig. 1(C)), despite the reported role of RhoA GTPase as a regulator of stress fibre formation.

### IFT88 mutation alters F-actin and NMIIB expression following cytochalasin D treatment

**Fig. 2:**
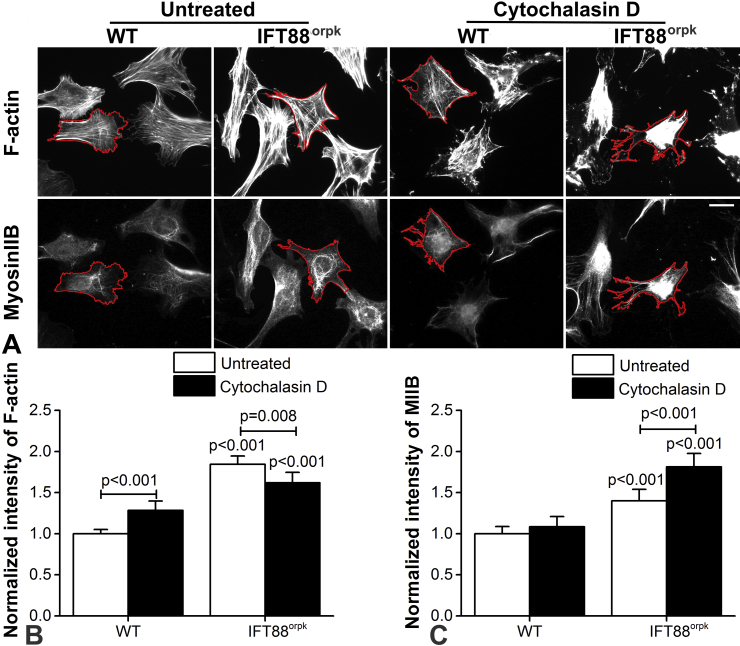
**IFT88 mutation increases disassembly of F-actin associated with increased myosin IIB expression with cytochalasin D treatment**. (A) Representative epifluorescence images showing the changes of F-actin (top panels) and myosin IIB (bottom panels) in WT and IFT88^orpk^ cells exposed to cytochalasin D (6 μM) for 10 min. F-actin was stained with Alexa Fluor 555-phalloidin while myosin IIB was labelled with antibodies targeted to NMIIB. Cell edges were identified by the red lines drawn around individual cells. Scale bar represents 25 μm. Changes in mean fluorescence intensity of (B) F-actin and (C) myosin IIB for WT and IFT88^orpk^ cells under each condition. Fluorescence intensity was normalized to the mean intensity of corresponding untreated WT cell groups. Data represents mean ± CI (*N* = 32 and 36). Data was analysed by unpaired Student's *t* test.

For both WT and IFT88^orpk^ chondrocytes, F-actin was disrupted at the cell periphery such that staining was completely lost, while aggregation or clumping of F-actin was observed in the perinuclear region [Fig. 2(A)]. Mean cellular F-actin intensity was significantly increased in WT chondrocytes after cytochalasin D treatment (*P* < 0.001; Fig. 2(B)). By contrast, IFT88^orpk^ chondrocytes exhibited a significant decrease in F-actin intensity after cytochalasin D treatment (*P* = 0.008; Fig. 2(B)). In WT cells, NMIIB remained relatively unchanged by cytochalasin D treatment. However, in IFT88^orpk^ chondrocytes cytochalasin D treatment significantly increased cellular NMIIB intensity (*P* < 0.001; Fig. 2(C)). IFT88^orpk^ cells continued to show a significantly greater level of F-actin and NMIIB relative to WT cells following cytochalasin D treatment (*P* < 0.001 in all cases; Fig. 2(B) and (C)).

### IFT88 mutation reduces cortical F-actin organisation in suspended chondrocytes

**Fig. 3:**
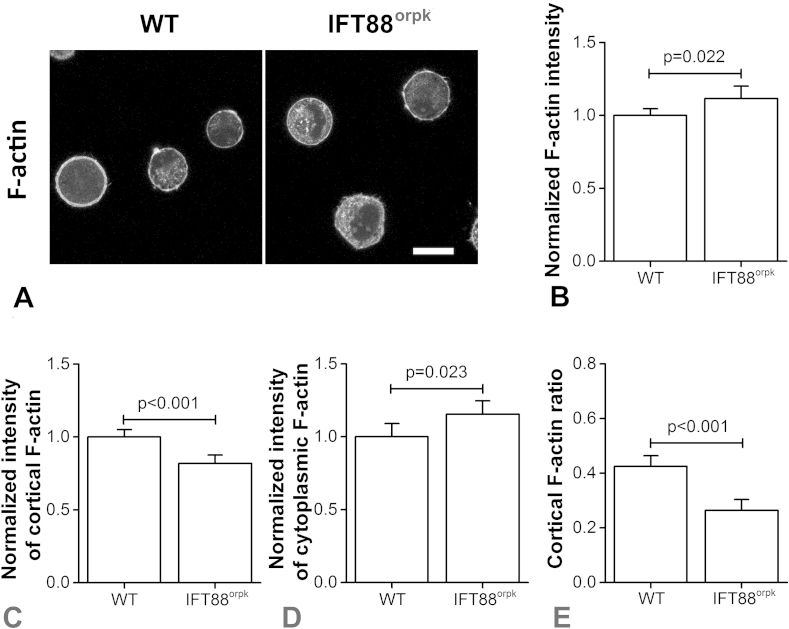
**IFT88 mutation decreases cortical F-actin expression**. (A) Representative confocal images showing F-actin stained with Alexa Fluor 555-phalloidin for WT and IFT88^orpk^ cells in suspension. Scale bar represents 15 μm. Quantification of (B) whole cell F-actin, (C) cortical F-actin, (D) cytoplasmic F-actin and (E) cortical F-actin ratio for WT and IFT88^orpk^ cells in suspension. Fluorescence intensities were normalised to the mean intensity of corresponding WT cell groups. Data represents mean ± CI (*N* = 71 and 94). Data was analysed by unpaired Student's *t* test.

In both WT and IFT88^orpk^ chondrocytes, the F-actin cytoskeleton was reorganised from stress fibres to cortical F-actin following 45 min in suspension culture reflecting the organisation *in situ* within articular cartilage^10^, ^37^ [Fig. 3(A)]. IFT88^orpk^ chondrocytes continued to exhibit greater expression of total F-actin compared to WT chondrocytes as reflected by a significant difference in the mean cellular intensity (*P* = 0.022; Fig. 3(A) and (B)). However, further quantitative analysis of the spatial distribution revealed that IFT88^orpk^ cells exhibited less cortical F-actin (*P* < 0.001; Fig. 3(C)) and more cytoplasmic F-actin (*P* = 0.023; Fig. 3(D)) compared to WT cells. This resulted in a significantly lower cortical F-actin ratio in IFT88^ORPK^ cells (*P* < 0.001; Fig. 3(E)) as adopted in previous studies^30^.

### IFT88 mutation reduces the equilibrium modulus of isolated chondrocytes

**Fig. 4:**
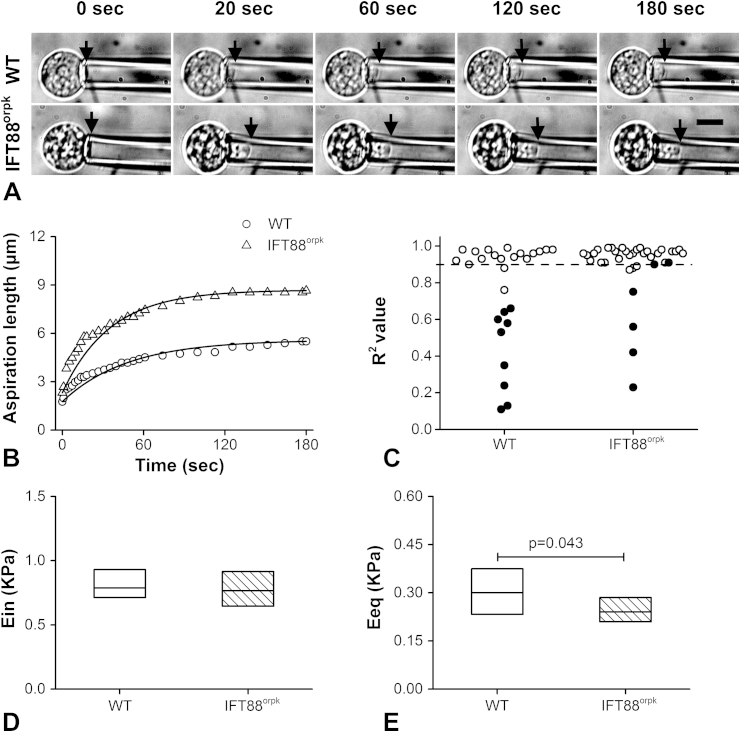
**IFT88 mutation decreases cell equilibrium modulus**. (A) Representative bright field images showing micropipette aspiration of individual WT and IFT88^orpk^ cells inside the pipette at 0 s, 20 s, 60 s, 120 s and 180 s after the application of 0.689 kPa pressure. Scale bar represents 10 μm. (B) Corresponding temporal changes in aspiration length fitted using the SLS model. (C) R^2^ value for SLS model curve fitting for all cells. Cells exhibiting retraction during aspiration are showing in black dots. (D) Instantaneous modulus and (E) equilibrium modulus were estimated using SLS model. Data represents median and quartiles (*N* = 16 and 29). Data was analysed by Mann–Whitney *U* test.

Micropipette aspiration was used to determine the viscoelastic properties of isolated cells and the effect of IFT88 mutation. Representative bright field images of individual WT and IFT88^orpk^ chondrocytes are shown along with associated temporal changes in aspirated length [Fig. 4(A) and (B)]. The majority of WT and IFT88^orpk^ chondrocytes exhibited viscoelastic creep behaviour in response to a step increase in pressure, such that cells were initially aspirated rapidly into micropipette followed by a decreased aspiration rate until reaching an equilibrium length. This response was accurately fitted using the SLS model (Table I and Fig. 4(B)). However, we observed that a small percentage of cells in both cell types showed initial elongation into the micropipette followed by retraction, which resulted poor model fitting R^2^ values less than 0.9 (retraction cells were shown as black dots; Fig. 4(C)). In terms of viscoelastic properties, no difference was found in the instantaneous modulus (*P* = 0.627; Fig. 4(D)), however the equilibrium modulus was significantly decreased in IFT88^orpk^ chondrocytes compared to WT chondrocytes (*P* = 0.043; Fig. 4(E)).

### IFT88 mutation reduces bleb formation and subsequent rate of actin remodelling

**Fig. 5:**
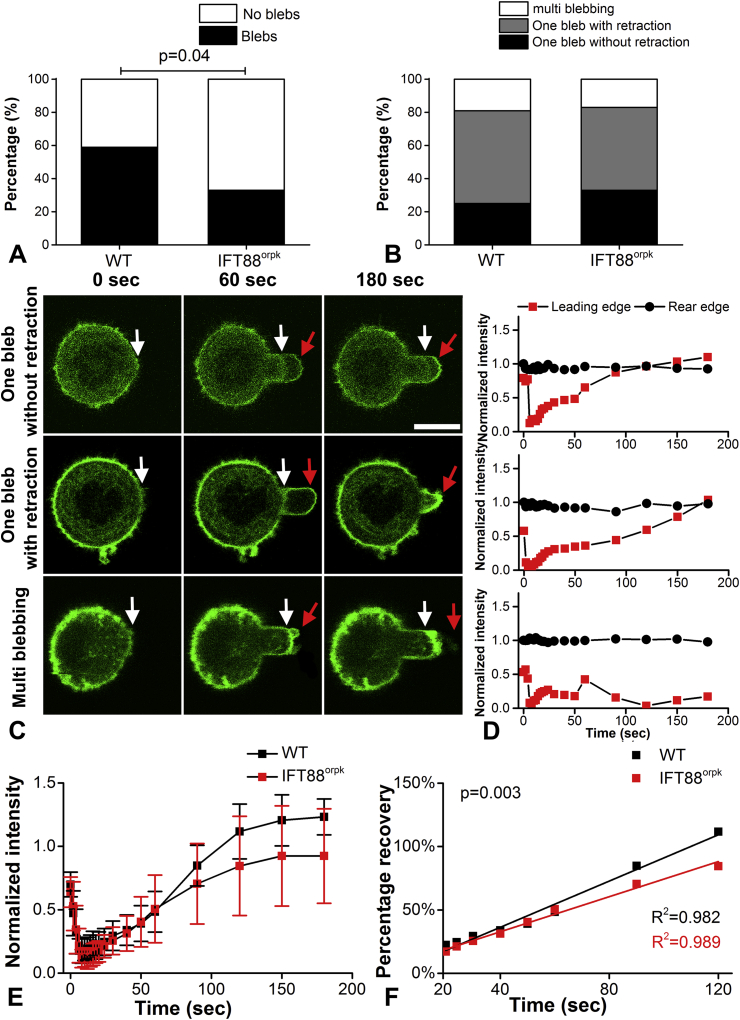
**IFT88**^**orpk**^**cells exhibit reduced blebbing and decreased rate of actin remodelling during micropipette aspiration**. Histograms showing (A) percentage of cells exhibiting blebbing and (B) three different classes of blebbing during aspiration in WT and IFT88^orpk^ cells. Data was analysed by Chi-square test (*N* = 27 and 36). (C) Representative confocal images showing aspirated cells exhibiting either one bleb without retraction, one bleb with retraction and multi blebbing with visualisation of cortical F-actin remodelling using LifeAct-GFP. White arrows indicate the initial F-actin cortex and red arrows for the newly formed cortex. Scale bar represents 10 μm. (D) Corresponding temporal changes in fluorescence intensity at the leading edge inside the pipette and the rear edge outside the pipette. The intensity of leading edge was normalised to the initial value at the rear edge. (E) The temporal changes in fluorescence intensity for cells showing one bleb during micropipette aspiration. Data represents mean and CI (*N* = 10 and 9). Data was analysed by unpaired Student's *t* test. (F) The rate of actin remodelling during micropipette aspiration. The mean intensity from 20 s to 120 s time points were fitted with linear regression. Univariate analysis of variance was used to examine the differences between two slopes.

It was noted that in some cells, micropipette aspiration resulted in the formation of membrane blebs in which the cell membrane detached from the cortex at the leading edge of the aspirated portion of the cell. This mechanically induced blebbing was significantly reduced in IFT88^orpk^ chondrocytes where 33% of cells exhibited blebs compared to 59% cells in WT cells (*P* = 0.04; Fig. 5(A)). Blebbing cells were classified into three groups based on whether cells showed: one bleb without retraction, one bleb with retraction or multiple blebs. There was no significant difference in the percentage of cells exhibiting each type of blebbing between two cell types (*P* = 0.691; Fig. 5(B)). To examine the cortical F-actin remodelling during bleb formation, cells were transduced with LifeAct-TagGFP2. In all three blebbing behaviours, a dramatic drop in cortical F-actin intensity occurred simultaneously with the bleb initiation in both cell types [Fig. 5(C) and (D)]. Cortical intensity at the leading edge of the bleb then gradually recovered back to the initial value as a new F-actin cortex reformed. No significant difference was found when comparing the fluorescence intensity at individual time points (*P* > 0.05 in all cases; Fig. 5(E)). However, the temporal changes in cortical F-actin intensity showed that IFT88^orpk^ chondrocytes had a significantly decreased rate of actin remodelling based on the gradients of linear model fitted to the data between 20 s and 120 s (*P* = 0.003; Fig. 5(F)). The small percentage of multi blebbing cells were not included in the analysis as it was difficult to accurately trace the F-actin cortex remodelling.

### IFT mutation reduces cortical tension and increases the deformability of the F-actin cortex

**Fig. 6:**
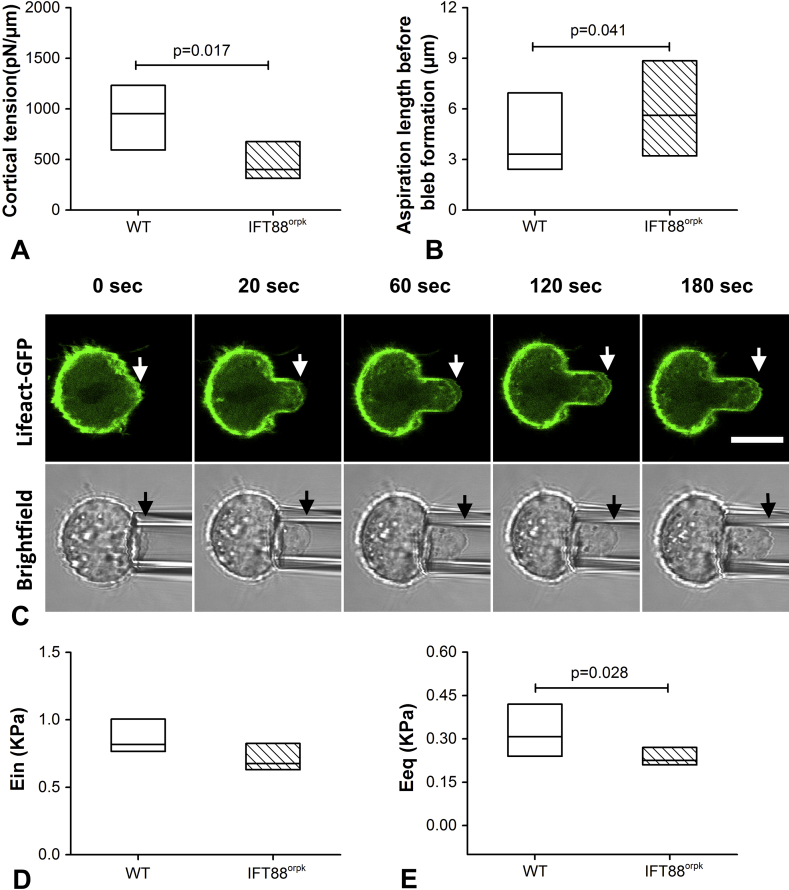
**IFT88**^**orpk**^**cells exhibit decreased cortical tension and increased deformability of F-actin cortex**. (A) IFT88^orpk^ cells exhibit decreased cortical tension compared with WT cells. Data represents median and quartiles (*N* = 20 and 17). Data was analysed by Mann–Whitney *U* test. (B) IFT88^orpk^ cells exhibit a significant increase in aspiration length before bleb formation. Data represents median and quartiles (*N* = 27 and 36). Data was analysed by Mann–Whitney *U* test. (C) Representative confocal images showing a LifeAct-GFP tagged cell exhibiting no blebs during aspiration. White arrows indicate the leading edge of cortical F-actin and black arrows for the leading edge of cell membrane. (D) Instantaneous modulus and (E) equilibrium modulus for cells exhibiting no blebs were estimated using SLS model. Data represents median and quartiles (*N* = 10 and 21). Data was analysed by Mann–Whitney *U* test.

To determine if IFT88 regulates actin mechanics independent of bleb formation, further studies were conducted to measure cortical tension using micropipette aspiration. This parameter effectively quantifies the mechanical properties of the F-actin cortex. IFT88^orpk^ chondrocytes exhibited a significantly decreased cortical tension compared to WT chondrocytes (*P* = 0.017; Fig. 6(A)). In order to investigate the deformability of cortical F-actin, we also measured the maximum aspiration length before bleb formation for those cells used in the measurement of viscoelastic properties. IFT88^orpk^ cells showed a significantly increased maximum aspiration length (*P* = 0.041; Fig. 6(B)). Finally, we used the SLS model to estimate the viscoelastic properties of non blebbing cells for which the cortical F-actin deformed in line with the whole cell [Fig. 6(C)]. In the absence of bleb formation, the instantaneous modulus of IFT88^orpk^ chondrocytes was still not significantly different compared to WT chondrocytes (*P* = 0.072; Fig. 6(D)) while the equilibrium modulus remained significantly lower (*P* = 0.028; Fig. 6(E)).

## Discussion

IFT88^orpk^ chondrocytes exhibited more pronounced F-actin stress fibres and greater mean staining intensity compared to WT chondrocytes in sub-confluent monolayer. In addition, our results demonstrate that IFT88^orpk^ cells exhibited increased expression of NMIIB indicative of altered intracellular tension^38^, ^39^. These results in chondrocytes differ from those reported for IFT88^orpk^ endothelial cells for which there was a reduction in stress fibre formation compared to WT^5^. However, other studies using renal medullary cells have shown that cilia BBS proteins increase F-actin stress fibre formation and that this is mediated by increased levels of RhoA-GTP^6^. The authors found that treatment with the RhoA inhibitors restored the actin cytoskeleton and prevented the reduction in primary cilia length in BBS-4 deficient cells. Surprisingly, our results showed no significant difference in RhoA activity between WT and IFT88^orpk^ chondrocytes, suggesting that other signalling pathways might be involved in the regulation of F-actin stress fibre assembly by IFT88. For example, previous studies suggest that in endothelial cells, IFT88 regulates stress fibre and focal adhesion assembly via a signalling pathway mediated by heat shock protein 27 (HSP27)^5^ which is present in chondrocyte primary cilia^40^. Other studies have revealed a novel signalling pathway in which the cilia protein polycystin-1 (PC1), which is disrupted in IFT88^orpk^ chondrocytes^41^, regulates actin organization through the PC1-Pacsin2-N-Wasp complex^42^.

Further studies were conducted to investigate the dynamics of F-actin stress fibres following treatment with cytochalasin D, as used in similar previous studies^6^. A low dose of cytochalasin D (6 μM, 10 min) was used to ensure the disruption of the F-actin stress fibres without major changes in cell morphology. After cytochalasin D treatment, F-actin intensity was significantly increased with the formation of F-actin aggregates in WT chondrocytes in agreement with previous studies using human leukocytes and murine B lymphocytes^43^, ^44^. Interestingly, this response was not observed in IFT88^orpk^ chondrocytes which may be due to altered regulation of actin dynamics and remodelling by intracellular Ca^2+^ which is disrupted in IFT88^orpk^ chondrocytes^27^. Another explanation might be that increased NMIIB in IFT88^orpk^ cells induces accelerated F-actin stress fibre disassembly, which is in agreement with *in vitro* studies showing NMIIB induces disassembly of actin stress fibres^45^.

Within articular cartilage and the developing growth plate, chondrocytes display a rounded morphology with a cortical F-actin organisation and no stress fibre bundles^10^, ^37^. Similar morphology and actin organisation is also observed *in vitro* in chondrocyte suspension culture^31^. In rounded cells, IFT88^orpk^ chondrocytes exhibited greater mean F-actin staining intensity compared to WT chondrocytes, consistent with that seen in monolayer. This agrees with previous studies which show increased F-actin staining in chondrocytes *in situ* within the presumptive articular cartilage and proliferative zones of the growth plate of IFT88^orpk^ mice^9^. In the present study, we show that IFT88^orpk^ chondrocytes in suspension exhibit reduced cortical F-actin staining compared to WT, despite the increase in overall mean staining intensity. These results agree closely with those of Song *et al.*, who examined the role of IFT in chondrocytes with deleted KIF3A, a subunit of the Kinesin II motor complex that is required for IFT^10^. They reported a reduction in cortical actin organisation in chondrocytes *in situ* within the post-natal growth plate of KIF3A null mice. Further studies^46^, ^47^, ^48^ have shown that the formation of cortical F-actin in cell suspension is regulated by Rho GTPase proteins especially RhoA, calmodulin and by myosin II-based contraction, all of which may be disrupted in IFT88^orpk^ chondrocytes.

To examine how alterations in F-actin organisation influence cell mechanics, micropipette aspiration in conjunction with the SLS model was used to estimate chondrocyte viscoelastic mechanical properties. Values for instantaneous and equilibrium moduli for WT cells match closely with those previously reported for primary articular chondrocytes using the same technique^31^, ^49^. IFT88^orpk^ chondrocytes were found to have significantly decreased equilibrium modulus compared to WT chondrocytes. In addition, the percentage of cells showing membrane blebs during aspiration was significantly reduced in IFT88^orpk^ cells. Since this may influence the measurement of the apparent cell modulus^50^, further studies were conducted to identify whether IFT88 influenced the mechanics of the actin cortex independent from bleb formation. In addition to the SLS model, these studies also used the liquid-drop model to determine the cortical tension as an indicator of the actin cortex mechanics. Although this model is most used for cells exhibiting fluid-like behaviour, it may also be applied to chondrocytes, including those used here with IFT88 disruption, which show solid-like viscoelastic behaviour^35^, ^50^, ^51^. Using cells transduced with LifeAct-TagGFP2, we show that IFT88^orpk^ chondrocytes exhibited reduced cortical F-actin remodelling following bleb formation, consistent with the disruption of actin remodelling following cytochalasin D treatment. Future work might be conducted to investigate the effects of blebbistatin, an inhibitor of myosin II, on actin organisation and bleb formation. IFT88^orpk^ chondrocytes also showed reduced retraction following bleb formation indicative of the slower actin remodelling^52^. In addition, IFT88^orpk^ chondrocytes exhibited significantly decreased cortical tension and an aspiration length before bleb formation which was significantly increased compared to WT. Similarly the viscoelastic properties calculated only for non blebbing cells also found that IFT88^orpk^ chondrocytes have significantly decreased equilibrium modulus. Together these results indicate that IFT88 influences the deformability and stiffness of the actin cortex, consistent with the reduced cortical tension and reduced cortical F-actin staining intensity. This change in the actin cortex in IFT88^orpk^ chondrocytes is thus responsible for the reduced effective whole cell equilibrium modulus and the reduced bleb formation which is associated with reduced intracellular pressure.

In summary, we show that IFT88 mutation results in alterations in chondrocyte actin-myosin stress fibre assembly and dynamics in response to cytochalasin D treatment. Moreover, we show that in rounded chondrocytes, IFT88 mutation reduces the formation and mechanical properties of the actin cortex. This in turn influences whole cell responses to mechanical stimuli including effective cellular mechanical properties and bleb formation. Our findings therefore demonstrate for the first time, the role of IFT in regulating fundamental actin organisation, dynamics and biomechanics. This provides new evidence identifying the role of primary cilia and IFT in genetic ciliopathies and in cartilage development, health and disease.

## Author contributions

Study conception and design: Zhao Wang, Angus KT. Wann, Clare L. Thompson, Martin M. Knight.

Acquisition of data: Zhao Wang, Clare L. Thompson, Aisha Hassen.

Analysis and interpretation of data: Zhao Wang, Angus KT. Wann, Clare L. Thompson, Wen Wang, Martin M. Knight.

## Competing interests

The authors declare no conflict of interest.

## Figures and Tables

**Table I:** The numbers of cells analysed for WT and IFT88^orpk^ cells including the numbers of cells rejected from the analysis and the ratio between cell diameter and micropipette diameter. Data is presented as median and interquartile

Cell type	Cell number			Cell diameter/pipette diameter
Total cells tested	Successfully aspirated	R^2^ > 0.9
WT	28	27 (96%)	16 (57%)	2.77 (0.25)
IFT88^orpk^	40	36 (90%)	29 (73%)	2.94 (0.39)
